# Deep-Sea Water Containing Selenium Provides Intestinal Protection against Duodenal Ulcers through the Upregulation of Bcl-2 and Thioredoxin Reductase 1

**DOI:** 10.1371/journal.pone.0096006

**Published:** 2014-07-01

**Authors:** Chih-Ching Yang, Chien-An Yao, Yi-Ruu Lin, Jyh-Chin Yang, Chiang-Ting Chien

**Affiliations:** 1 Department of Planning, Ministry of Health and Welfare, Executive Yuan, Taipei, Taiwan; 2 Department of Internal Medicine, National Yang-Ming University, Taipei, Taiwan; 3 Department of Family Medicine, Hospital and College of Medicine, National Taiwan University, Taipei, Taiwan; 4 Water Resources Division, Stone and Resource Industry Research and Development Center, Hualien, Taiwan; 5 Department of Internal Medicine, Hospital and College of Medicine, National Taiwan University, Taipei, Taiwan; 6 Department of Life Sciences, National Taiwan Normal University, Taipei, Taiwan; Institute of Biomedical Sciences, Taiwan

## Abstract

Deep-sea water (DSW), which is rich in micronutrients and minerals and with antioxidant and anti-inflammatory qualities, may be developed as marine drugs to provide intestinal protection against duodenal ulcers. We determined several characteristics in the modified DSW. We explored duodenal pressure, oxygenation, microvascular blood flow, and changes in pH and oxidative redox potential (ORP) values within the stomach and duodenum in response to tap water (TW, hardness: 2.48 ppm), DSW600 (hardness: 600 ppm), and DSW1200 (hardness: 1200 ppm) in Wistar rats and analyzed oxidative stress and apoptosis gene expressions by cDNA and RNA microarrays in the duodenal epithelium. We compared the effects of drinking DSW, MgCl_2_, and selenium water on duodenal ulcers using pathologic scoring, immunohistochemical analysis, and Western blotting. Our results showed DSW has a higher pH value, lower ORP value, higher scavenging H_2_O_2_ and HOCl activity, higher Mg^2+^ concentrations, and micronutrients selenium compared with TW samples. Water infusion significantly increased intestinal pressure, O_2_ levels, and microvascular blood flow in DSW and TW groups. Microarray showed DSW600, DSW1200, selenium water upregulated antioxidant and anti-apoptotic genes and downregulated pro-apoptotic gene expression compared with the TW group. Drinking DSW600, DSW1200, and selenium water but not Mg^2+^ water significantly enhanced Bcl-2 and thioredoxin reductase 1 expression. Bax/Bcl-2/caspase 3/poly-(ADP-ribose)-polymerase signaling was activated during the pathogenesis of duodenal ulceration. DSW drinking reduced ulcer area as well as apoptotic signaling in acetic acid-induced duodenal ulcers. DSW, which contains selenium, provides intestinal protection against duodenal ulcers through the upregulation of Bcl-2 and thioredoxin reductase 1.

## Introduction

Deep sea water (DSW) obtained from 200 m under the surface of the sea is characterized by high purity, low temperature, and high nutrient and mineral concentrations [1]–[3]. DSW has been used to reduce lipid profiles and lipid peroxidation [2]–[4], prevent mild hypertension and atherosclerosis [3], and decrease the accumulation of inflammatory foam cells in the aorta of dietary-induced hyperlipidemia in rabbits [5]. Intake of dissolved organic matter in DSW has been shown to inhibit neointimal hyperplasia in balloon-injured common carotid arteries [6]. In the development of atopic dermatitis, a type of chronic inflammatory skin disease, DSW ingestion or bathing improves dermatitis symptoms and allergic skin responses by reducing the inflammatory cell infiltration, and inhibiting the upregulation of IgE, histamine, and pro-inflammatory cytokines in the serum [7]–[8]. DSW intake has been shown to delay cataract development by decreasing nitric oxide levels in the lens of the shumiya cataract rat [9]–[10]. These data suggest that DSW may be helpful in the prevention and treatment of oxidative stress- and inflammation-related diseases.

Increased production of toxic reactive oxygen species (ROS) evokes abnormal signal transduction, cellular dysfunction, inflammatory monocyte/macrophage infiltration, and cell death cascade in the damaged tissues [11]–[13]. This enhanced oxidative stress may induce apoptotic cell death due to the increase in Bax/Bcl-2 ratio, the opening of the mitochondria permeability transition pore, and the release of mitochondrial cytochrome C into the cytosol, which increases caspase 3 activity and poly-(ADP-ribose)-polymerase (PARP) cleavage [11]–[13]. Bcl-2 family members have been reported to play a critical role in the regulation of *Helicobacter pylori* (*H. pylori*) infection-induced gastric apoptosis [13]. In duodenal ulcer patients infected with *H. pylori*, duodenal ulcers have been shown to be associated with a high degree of apoptosis [14]. We hypothesized that Bax/Bcl-2/caspase 3/PARP signaling may be involved in the pathogenesis of duodenal ulceration.

Oxidative stress and inflammation contribute to *H. pylori* infection and other factors that are known to induce gastrointestinal ulceration [15]. High intake of Mg^2+^ ions can improve *H. pylori* infection, gastric ulcer [16]–[18], and gastro-esophageal reflux [19]. Deficiencies in antioxidant vitamins and selenium can cause *H. pylori*-associated active chronic gastritis rapidly converting to chronic atrophic gastritis [20]. To date, the effect of antioxidant and anti-inflammatory DSW ingestion on duodenal ulcers has not been determined. We aimed to evaluate whether DSW ingestion has any beneficial effects for preventing acetic acid-induced duodenal ulcers in a rat model. We also compared the effect of MgCl_2_ and selenium on acetic acid-induced duodenal ulcers. Our data found that DSW ingestion may attenuate acetic acid-enhanced duodenal ulceration and apoptosis formation via action of selenium. DSW ingestion in daily life may have preventive or therapeutic potential for gastrointestinal ulceration.

## Materials and Methods

### Ethics statement

Original DSW was obtained from a depth of approximately 618 m in Chisingtan Bay, Hua-Lien County, Taiwan and was permitted by Stone and Resource Industry Research and Development Center (Guanghuajian, Hualien, Taiwan). Female Wistar rats (220–250 g in weight each) were purchased from BioLASCO Taiwan Co. Ltd. (Taipei) and housed at the Experimental Animal Center, National Taiwan Normal University, at a constant temperature and with a consistent light cycle (light from 0700 to 1800 h). The animal care and experimental protocols were approved by the National Taiwan Normal University and were in accordance with the guidelines of the National Science Council of the Republic of China (NSC 1997).

### DSW preparation

Modified DSW samples were prepared and provided by Stone and Resource Industry Research and Development Center (Guanghuajian, Hualien, Taiwan). In brief, the original DSW was processed by reverse osmosis (RO) and electrodialysis (ED) to reduce the sodium content. The hardness of DSW indicated in ppm was calculated by the following formula: [CaCO_3_] ppm = ([Ca^2+^]×2.5+[Mg^2+^]×4.1) ppm. After RO and ED, DSW drinking waters with hardness of 600 and 1200 ppm were obtained. DSW drinking waters were pasteurized at 80°C for 60 s and immediately stored at room temperature (25°C) until given to the test animals. The mineral contents in each sample of water were analyzed using an inductively coupled plasma optical emission spectrometer (JY ULTIMA 2000, Horiba, France). The pH value, hardness, and major mineral concentrations (including selenium) of each drinking water (2.1 ppm tap water [TW], 589 ppm deep-sea water drinking water [DSW600], and 1185 ppm DSW drinking water [DSW1200]) are shown in **Table 1**.

**Table 1:** The characteristics of pH, ORP, antioxidant activity, and mineral contents of the different types of water studied.

	TW	DSW600	DSW1200
pH	6.68	7.01	7.29
ORP (mV)	344	203	125
H_2_O_2_-CL counts (counts/10 s)	1.20±0.42 (×10^7^)	5.82±1.90 (×10^6^)	1.72±0.54 (×10^6^)
HOCl CL counts(counts/10 µs)	3.11±0.62 (×10^5^)	1.09±0.34 (×10^5^)	5.21±1.63 (×10^4^)
Na (mg/dL)	0.45	368	738
K (mg/dL)	0.11	67	133
Ca (mg/dL)	0.21	1.02	2.08
Mg (mg/dL)	0.35	225	448
Mg/Ca	1.66	220	215
Hardness (ppm)	2.1	589	1185
Selenium (µg/L)	ND	0.0011	0.0023
As (µg/L)	ND	ND	ND
Cr (µg/L)	ND	ND	ND
Ba (µg/L)	ND	ND	ND
Cd (µg/L)	ND	ND	ND
Pb (µg/L)	ND	ND	ND
Hg (µg/L)	ND	ND	ND

ND: not detectable in our samples.

### Measurement of pH and ORP values and antioxidant activity

We measured the oxidative redox potential (ORP) values (HI 8014, portable pH/ORP meter, HANNA Instruments, Cluj-Napoca, Romania) and pH values (Denver Instrument UltraBasic UB-10 pH Benchtop meter, Cole-Parmer, Vernon Hills, Illinois, USA) of the samples. We determined the scavenging H_2_O_2_ and HOCl activity of the samples using a modified luminol-ultrasensitive chemiluminescence assay [21].

### Animal surgery

The rats were anesthetized with subcutaneous urethane (1.2 g/kg). Body temperature was maintained at 36.5–37.0°C by an infrared light and was monitored with a rectal thermometer. PE50 catheters were placed in the left carotid artery to measure heart rate and arterial blood pressure (ABP) using an ADI system (PowerLab/16S, ADI Instruments, Pty Ltd, Castle Hill, Australia) with a transducer (P23 1D, Gould-Statham, Quincy, CA), and in the left femoral vein for administration of tested drugs or anesthetics when needed.

### Characteristics of water in the stomach and intestine

**Figure 1:**
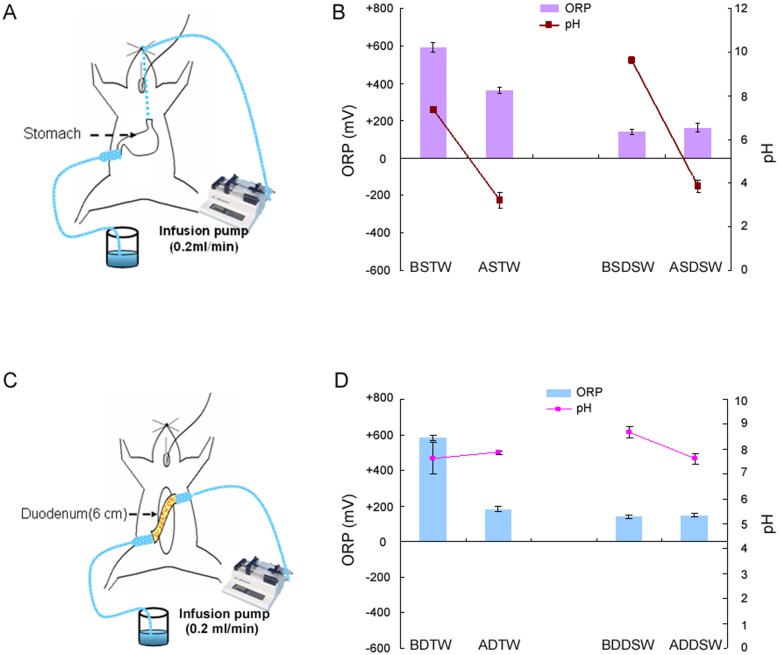
The characteristics of pH and ORP values of water solution before and after the stomach and duodenum. The setup for the measurement of changes in pH and ORP values of the tap water (TW) or deep-sea water (DSW) solution after passing through the stomach (A) or duodenum (C). The statistical data relating to pH and ORP values before and after stomach or duodenum pathway are displayed in B and D, respectively. BSTW = TW before passing through the stomach; ASTW = TW after passing through the stomach; BSDSW = DSW before passing through the stomach; ASDSW = DSW after passing through the stomach; BDTW = TW before passing through the duodenum; ADTW = TW after passing through the duodenum; BDDSW = DSW before passing through the duodenum; ADDSW = DSW after passing through the duodenum.

To measure the optimal pH and ORP values of the waters in the stomach, a PE200 tube was inserted to the esophagocardiac junction of the stomach via the oral cavity and different waters were infused into the stomach. The waters were infused at a rate of 0.2 mL/min for 60 min. Another PE200 tube was inserted from the duodenal to the pyloric area to collect the waters in order to measure changes in ORP and pH values in the stomach (**Figure 1A**).

To measure optimal pH and ORP values of the waters in the duodenum, a PE200 tube was inserted into the duodenum from the pyloric stomach (**Figure 1C**) and different waters were infused into the duodenum at a rate of 0.2 mL/min for 60 min. Another PE200 tube was inserted from the distal end of the duodenal area to collect the waters in order to measure changes in ORP and pH values.

### cDNA microarray analysis

A detailed protocol for rat cDNA microarray analysis has been reported previously [22]. Epithelial cells were isolated from the small intestine of the rats [23]. In brief, the small intestine was rapidly removed and washed with ice-cold saline solution. Proximal intestinal segments (1.0 cm) were incubated at room temperature in phosphate buffer saline (PBS) containing 1 mM dithiothreitol for 15 min, followed by a 30-min incubation period at 37°C in a calcium- and magnesium-free PBS containing 1 mM EDTA and 2 mM glucose. Following the incubation, the tissues were vortexed for 30 s, the loosened epithelial cells were filtered through a 60-µm nylon textile and collected by centrifugation and resuspension in PBS. RNA from isolated intestinal cells was treated with Trizol reagents and quality control was determined using a NanoDrop ND-1000 spectrophotometer (Thermo Scientific). Five micrograms of mRNA from each sample was used with commercial [Rat230_2] Affymetrix Rat Genome 230 2.0 Array and was performed in National Taiwan University Microarray Core. Affymetrix submissions are typically submitted to *Gene Expression Omnibus* (GEO) using the GEOarchive method and obtained the accession number (GSE55142).

### RNA purification microarrays

The preparation of RNA with RNase-Free DNase Set (Qiagen) and RNeasy MinElute Cleanup Kit (Qiagen) was analyzed with SABioScience RT^2^ Profiler Q-PCR array. PCR reactions were performed to evaluate the expression of 84 genes using Qiagen RT^2^ Prolifer PCR array PARN-012ZA (Rat Apoptosis SuperArray) and PARN-065ZA (Rat Oxidative Stress SuperArray). Real-time PCR was performed on ABI Prism 7900 HT (Applied Biosystems) according to the manufacturer’s instructions, and analyzed using ViiA-7 Real-Time PCR System. The C_t_ values were measured by using the web-based PCR array data analysis software.

### Measurement of duodenal blood flow and tissue oxygen tension

Duodenal pressure, microcirculation, tissue oxygenation, and temperature were evaluated simultaneously using PE50 tubing connected to a pressure transducer and one PO_2_/temperature/perfusion sensor-containing single fiber optic + thermocouple + laser Doppler fiber and recorded with a recording system (OxyLite 2000E+OxyFlo, Oxford Optronix Limited, Oxford, UK) [24].

At the end of each experiment, the rats were sacrificed with an overdose of anesthetics. The duodena were removed and divided into two parts. One part was stored in 10% neutral buffered formalin for pathologic and immunohistochemical assays, and the other was stored at −80°C for further analysis.

### Duodenal ulcer induction

DSW drinking at hardness of 200–1000 ppm (Mg^2+^200–1000 mg/L) delayed cataract development [9], [10], at hardness of 1200 pm (Mg^2+^241 mg/L) [5] or 1400 ppm (Mg^2+^386 mg/L) [4] decreased serum total and low-density lipoprotein-cholesterol and prevented hyperlipidemia and arteriosclerosis. Therefore, we prepared DSW with a lower hardness of 600 ppm (DSW600) and a higher hardness of 1200 ppm (DSW1200) in this study. We tested acetic acid-induced duodenal ulcers in the TW, DSW600, DSW1200, magnesium chloride (MgCl_2_) water, and selenium water groups (n = 6 each). We used the guideline for conversion of animal doses to human equivalent doses based on body surface area (Guidance for Industry Estimating the Maximum Safe Starting Dose in Initial Clinical Trials for therapeutics in Adult Healthy Volunteers, US Department of Health and Human Services Food and Drug Administration Center for Drug Evaluation and Research 2005, Pharmacology and Toxicology). Referring to the human recommended daily allowance (RDA) of magnesium at 350 mg/day [10], these rats drank 41±3 mL/day of DSW600 (equivalent to 94.5 mg Mg^2+^/day) or 39±3 mL/day of DSW1200 (equivalent to 176.1 mg Mg^2+^/day). The dosage of DSW1200 (equivalent to 176.1 mg Mg^2+^/day) was estimated as following: 350 mg/day (a maximum recommended starting dose in human) ÷ 60 (kg/body weight) ×6.2 (the conversion factors for converting human equivalent dose in mg/kg to animal dose in mg/kg) ×5 (safety factor) = 180 mg Mg^2+^/day in the rats. Additionally, MgCl_2_ water (225 and 450 mg/dL, Sigma-Aldrich) or selenium water treatment was conducted for comparison with the DSW supplement. We prepared MgCl_2_ water at 225 and 450 mg/dL for production of similar Mg^2+^ level like DSW600 and DSW1200 to evaluate Mg^2+^ effect on acetic acid-induced duodenal ulcer. Because selenium is contained in DSW (0.005–5 µg/L) [2], [9] and induced anti-apoptotic potential at concentration of 1.5 mg/L of sodium selenite [25], we therefore used sodium selenite (Sigma-Aldrich, 1 mg/L of distilled H_2_O) to test its effect on acetic acid-induced ulcer and apoptosis. These rats drank 42±4 mL of 225 mg/dL of MgCl_2_ water (equivalent to 94.5 mg Mg^2+^/day), 40±4 mL of 450 mg/dL of MgCl_2_ water (equivalent to 180 mg Mg^2+^/day), or 39±4 mL of selenium water (equivalent to 0 mg Mg^2+^/day and selenium 39 µg/day).

The abdomen was opened and the duodenum was exposed. A plastic tube, 4.2-mm diameter, was applied tightly to the wall of the duodenum approximately 5 mm beyond the pylorus. Approximately 70 µL of 100% acetic acid was applied for 10 s to the mucosa surface of the duodenum. After removal of the acetic acid, the abdomen was sutured. This resulted in the formation of ulcers of the mucosa and submucosa within the area of acetic acid application. All the rats were fasted with unlimited access to tested water under the day of acetic acid induction and then had free access to food and tested waters after recovery. We only examined acetic acid-induced duodenal ulcer and apoptosis after 24 hours (Day 1) or 72 hours (Day 3) of acetic acid stimulation. Twenty-four hours of water restriction (WR) would damage duodenal epithelium according to our microarray information, therefore, we did not use acetic acid-induced ulcer in the WR group in the present study. At the end of each experiment i.e. after either Day 1 or Day 3 of ulcer induction, the animals were sacrificed with an intra-peritoneal overdose of anesthetics.

### Western blotting

Expression levels of apoptosis-related proteins including Bcl-2 (Transduction, Bluegrass-Lexington, KY), Bax (Chemicon, Temecula, CA), caspase 3 (CPP32, Upstate Biotechnology, Lake Placid, NY), PARP (Cell Signaling Technology, Inc., Danvers, MA), and β-actin (Sigma, Saint Louis, MI) were analyzed using Western blotting in the duodenal epithelium from the ulcers of rats in the different groups [24].

### Pathology and TUNEL immunohistochemistry

The histology and histological scoring of duodenal sections for acetic acid-induced ulcers were determined as described previously [26]. At the indicated time, all animals were sacrificed and the duodena were excised carefully and cut open along the anti-mesenteric side. The duodenal ulcer was developed on the anti-anterior wall (anti-mesenteric wall) of the duodenum. The photographs of the duodenum were digitized and converted to binary images through gray scale imaging. Using the National Institute of Health (NIH) image-J software, the area of duodenal ulcers (mm^2^) was calculated. The percentage area was calculated using the following formula:
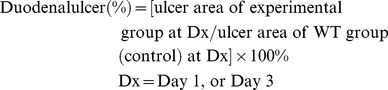



Increased oxidative injury may contribute to apoptosis. We performed terminal deoxynucleotidyl transferase-mediated nick-end labeling (TUNEL) for evaluating apoptosis in the paraffin-embedded sections of duodenal tissues [11]. Briefly, 5-µm thick sections of duodenal tissues were prepared, deparaffinized, and stained by the TUNEL-ABC method. A biotinylated secondary antibody (Dako, Botany, NSW, Australia) was then applied followed by streptavidin conjugated to HRP (Dako). The chromogen used was Dako Liquid diaminobenzene. Twenty high-powered (×400) fields of the sections were randomly selected in each section, and the number of apoptotic cells was counted. The value of apoptotic cells/(apoptotic cells and methyl green stained cells in the duodenal cells) was calculated in high-powered (×400) fields. For ulcer and apoptosis detection, we only included the duodenal tissues with tested waters after 24 hours (Day 1) or 72 hours (Day 3), respectively. TW drinking was selected as control group in the present study. We did not adapt the rats without water drinking for 24 hours. All histological sections were analyzed using a Sonix Image Setup (Sonix Technology Co., Ltd) containing image analyzing software Carl Zeiss AxioVision Rel.4.8.2 (Future Optics & Tech. Co. Ltd., Hangzhou, China).

### Bacterial strains and the anti-bacterial activity of DSW

The method for evaluating anti-bacterial (*H. pylori*) activity of DSW was performed as described previously [12]. The bacterial strains from 10 clinical isolates of *H. pylori* were used. The clinical isolates were obtained from gastric biopsy specimens from patients with peptic ulcer after getting the informed consents. The protocol has been approved by the Clinical trial/Research Approval of National Taiwan University Hospital (NTUH-REC No.: 201304065RIND). The blood agar plate was prepared by the Brain Heart Infusion (BHI) agar with 10% sheep blood, 1% IsoVitalex, and antibiotics. The blood agar plates with the autoclaved DSW were prepared to the different hardness of 600, 1200 and 2400 ppm. *H. pylori* were cultured on the blood agar plates at 37°C under microaerophilic conditions (5% O_2_, 10% CO_2_, 85% N_2_) in duplicate. After 3–5 days, the situation of bacterial growth was observed and analyzed in the agar plates.

### Statistical analyses

All values are expressed as mean ± standard error mean (SEM). Between-group comparisons in characteristics of tested waters, duodenal ulcer area, apoptotic number, protein expression and anti-bacterial activity were performed using unpaired *t-*tests or analysis of variance with Bonferroni method as *post hoc* analysis. Within-group comparisons in characteristics of tested waters, duodenal ulcer area, apoptotic number, protein expression and anti-bacterial activity were performed using paired *t-*tests or repeated-measures analysis of variance with Bonferroni method as *post hoc* analysis. P-values of <0.05 were considered to be statistically significant. All calculations were performed using SPSS for WINDOWS software (version 13.0; SPSS Inc, Chicago, IL). All microarray data were normalized by quantile normalization. The statistics significance from microarray data was filtered with a 2-fold changed selection criterion from the corresponding genes.

## Results

### Characteristics of DSW

As shown in **Table 1**, DSW is characterized as having higher pH value, lower ORP value, higher Na^+^, K^+^, Ca^2+^, Mg^2+^ concentrations, and higher selenium micronutrients. In all, these components displayed higher antioxidant H_2_O_2_ and HOCl activity in terms of scavenging H_2_O_2_ and HOCl when compared with TW samples.

### Oxidative stress gene expression in response to DSW

To characterize signaling events underlying the different effects of water ingestion on the duodenal epithelium, we analyzed the activation of several transcription factors. Changes in oxidative stress gene expression differed significantly between the DSW, water restriction for 12 hours, and TW groups (*P*<0.05). These changes are shown in **Table 2**. Of the 84 oxidative stress genes that were evaluated, six were downregulated in the WR group; 4 were upregulated in the DSW600 group; and 20 were upregulated in the DSW1200 group. DSW1200 activates the expression of flavin-containing monooxygenase 2 (*Fmo2*), glutathione peroxidase 1, 5, 6 (*Gpx1, Gpx5, Gpx6*), glutathione reductase (*Gsr*), nitric oxide synthase 2, inducible *(Nos2)*, thioredoxin reductase 1 (*Txnrd1)*, superoxide dismutase 1 (*Sod1*), some antioxidant-related genes peroxiredoxin 4 (*Prdx4*), and selenoprotein P, plasma, 1 (*Sepp1*). DSW600 upregulates one antioxidant gene, DSW1200 upregulates 8 antioxidant genes, whereas TW upregulates none antioxidant genes, indicating the potential of DWS1200 as an antioxidant. SeW treatment upregulated 31 gene expressions and downregulated 17 genes expression in the oxidative stress related genes. Highly expressed antioxidant genes like *Gpx1* (+400%), *Gpx2* (+505%), *Gpx4* (+280%), *Gsr* (+212%), *Prdx5* (+273%), *Prdx6* (+800%), *Sepp1* (+216%), *Txnip* (+656%) *and Txnrd1* (+461%) were found in the rat intestinal cells vs. TW group.

**Table 2:** Expression of oxidative stress genes in the duodenal epithelium after water restriction (WR), DSW intake and TW intake.

Gene	Description	Fold Up- or Down-regulation
**WR vs. TW**		
*Fmo2*	Flavin containing monooxygenase 2	−2.66
*Fth1*	Ferritin, heavy polypeptide 1	−1.71
*Gsr*	Glutathione reductase	−1.55
*Prdx4*	Peroxiredoxin 4	−2.11
*Psmb5*	Proteasome (prosome, macropain) subunit, beta type 5	−1.62
*Sod1*	Superoxide dismutase 1, soluble	−2.09
**600DSW vs. TW**		
*Park7*	Parkinson disease (autosomal recessive, early onset) 7	+2.65
*Prnp*	Prion protein	+2.58
*Srxn1*	Sulfiredoxin 1 homolog (*S. cerevisiae*)	+2.15
*Txnrd1*	Thioredoxin reductase 1	+1.73
**1200DSW vs TW**		
*Apoe*	Apolipoprotein E	+4.45
*Dnm2*	Dynamin 2	+3.86
*Duox1*	Dual oxidase 1	+3.02
*Ehd2*	EH-domain containing 2	+3.87
*Ercc2*	Excision repair cross-complementing rodent repair deficiency, complementation group 2	+5.97
*Fancc*	Fanconi anemia, complementation group C	+5.29
*Fmo2*	Flavin containing monooxygenase 2	+4.11
*Gpx1*	Glutathione peroxidase 1	+3.67
*Gpx5*	Glutathione peroxidase 5	+3.33
*Gpx6*	Glutathione peroxidase 6	+3.91
*Gsr*	Glutathione reductase	+3.81
*Ngb*	Neuroglobin	+4.06
*Nos2*	Nitric oxide synthase 2, inducible	+3.01
*Park7*	Parkinson disease (autosomal recessive, early onset) 7	+3.99
*Prdx4*	Peroxiredoxin 4	+3.04
*Prnp*	Peroxiredoxin 4	+2.70
*Sepp1*	Selenoprotein P, plasma, 1	+2.60
*Srxn1*	Sulfiredoxin 1 homolog (S. cerevisiae)	+2.77
*Txnip*	Thioredoxin interacting protein	+2.22
*Txnrd1*	Thioredoxin reductase 1	+2.61
**SeW vs TW**		
*Als2*	Amyotrophic lateral sclerosis 2 (juvenile) homolog (human)	+1.51
*Apc*	Adenomatous polyposis coli	−1.12
*Cat*	Catalase	+1.62
*Ccl5*	Chemokine (C-C motif) ligand 5	−5.63
*Ccs*	Copper chaperone for superoxide dismutase	+3.32
*Ctsb*	Cathepsin B	+1.13
*Cyba*	Cytochrome b-245, alpha polypeptide	+5.48
*Dhcr24*	24-dehydrocholesterol reductase	+5.56
*Dnm2*	Dynamin 2	+6.34
*Ercc6*	Excision repair cross-complementing rodent repair deficiency, complementation group 2	−2.19
*Fth1*	Ferritin, heavy polypeptide 1	−1.98
*Gclc*	Glutamate-cysteine ligase, catalytic subunit	+1.37
*Gclm*	Glutamate cysteine ligase, modifier subunit	+1.47
*Gpx1*	Glutathione peroxidase 1	+4.00
*Gpx2*	Glutathione peroxidase 2	+5.05
*Gpx3*	Glutathione peroxidase 3	−1.18
*Gpx4*	Glutathione peroxidase 4	+2.80
*Gsr*	Glutathione reductase	+2.12
*Gstk1*	Glutathione S-transferase kappa 1	+1.39
*Gstp1*	Glutathione S-transferase pi 1	+1.59
*Hba-a2*	Hemoglobin alpha, adult chain 2	+1.02
*Hmox1*	Heme oxygenase (decycling) 1	−2.55
*Idh1*	Isocitrate dehydrogenase 1 (NADP+), soluble	+1.56
*Noxa1*	NADPH oxidase activator 1	−1.22
*Nqo1*	NAD(P)H dehydrogenase, quinone 1	+1.49
*Park7*	Parkinson disease (autosomal recessive, early onset) 7	+2.23
*Prdx1*	Peroxiredoxin 1	−2.96
*Prdx2*	Peroxiredoxin 2	−1.62
*Prdx3*	Peroxiredoxin 3	−1.28
*Prdx4*	Peroxiredoxin 4	−1.49
*Prdx5*	Peroxiredoxin 5	+2.73
*Prdx6*	Peroxiredoxin 6	+8.00
*Prnp*	Prion protein	+2.57
*Psmb5*	Proteasome (prosome, macropain) subunit, beta type 5	−1.47
*Ptgs1*	Prostaglandin-endoperoxide synthase 1	−1.12
*Sels*	Selenoprotein S	−2.30
*Sepp1*	Selenoprotein P, plasma, 1	+2.16
*Serpinb1b*	Serine (or cysteine) peptidase inhibitor, clade B, member 1b	−3.03
*Slc38a1*	Solute carrier family 38, member 1	+2.97
*SOD1*	Superoxide dismutase 1, soluble	−1.49
*SOD2*	Superoxide dismutase 2, mitochondrial	+1.78
*Sqstm1*	Sequestosome 1	+9.79
*Srxn1*	Sulfiredoxin 1 homolog (S. cerevisiae)	+2.62
*Txn1*	Thioredoxin 1	−3.32
*Txnip*	Thioredoxin interacting protein	+6.56
*Txnrd1*	Thioredoxin reductase 1	+4.61
*Txnrd2*	Thioredoxin reductase 2	−1.73
*Ucp2*	Uncoupling protein 2 (mitochondrial, proton carrier)	+13.03

**Fold-Change** [2∧(−Delta Delta Ct)] is the normalized gene expression [2∧(−Delta Ct)] in the test sample divided by the normalized gene expression [2∧(−Delta Ct)] in the control sample.

### Apoptotic gene expression in response to DSW

Of the 84 genes evaluated, the data in **Table 3** shows that 12 genes were upregulated and 1 was downregulated in the WR group compared with the TW group. DSW600 ingestion upregulated 4 genes and downregulated 7 genes in the duodenal epithelium compared with the TW group. DSW1200 ingestion upregulated 3 genes and downregulated 7 genes compared with the TW group. SeW ingestion upregulated 12 genes compared with the TW group. Several important genes involved in the initiation or activation of apoptotic signaling pathways were significantly upregulated in the WR group compared with the TW group, including: apoptosis-inducing factor, mitochondrion-associated 1 (*Aifm1*), Bcl2-associated athanogene (*Api5*), Bcl2-associated agonist of cell death (*Bad*), Bcl2-associated X protein (*Bax*), caspase 1 (*Casp1*), Cell death-inducing DFFA-like effector b (*Cideb*), and Fas (TNF receptor superfamily, member 6) (*Fas*). DSW600 or DSW1200 ingestion seems to downregulate more apoptosis-related genes expression than TW treatment, indicating its anti-apoptotic action. SeW treatment downregulated apoptotic genes expression as described above and upregulated several anti-apoptotic genes expression like *Mcl1* in the intestinal cells.

**Table 3:** Expression of apoptotic genes in the duodenal epithelium after water restriction, intake of DSW waters vs intake of TW.

Gene	Description	Fold Up- or Down-regulation
**WR vs. TW**		
*Aifm1*	Apoptosis-inducing factor, mitochondrion-associated 1	+1.64
*Api5*	Apoptosis inhibitor 5	+1.69
*Bag1*	BCL2-associated athanogene	+1.45
*Bad*	BCL2-associated agonist of cell death	+1.24
*Bax*	Bcl2-associated X protein	+1.55
*Casp1*	Caspase 1	+1.24
*Cideb*	Cell death-inducing DFFA-like effector b	+2.26
*Cycs*	Cytochrome c, somatic	+3.52
*Fas*	Fas (TNF receptor superfamily, member 6)	+1.70
*Gadd45a*	Growth arrest and DNA-damage-inducible, alpha	−1.12
*Mapk1*	Mitogen activated protein kinase 1	+1.34
*Pycard*	PYD and CARD domain containing	+2.03
*Xiap*	X-linked inhibitor of apoptosis	+1.36
**600DSW vs TW**		
*Aifm1*	Apoptosis-inducing factor, mitochondrion-associated 1	+1.25
*Api5*	Apoptosis inhibitor 5	−1.14
*Bag1*	BCL2-associated athanogene	−1.15
*Ciedb*	Cell death-inducing DFFA-like effector b	−1.32
*Cycs*	Cytochrome c, somatic	−1.59
*Fas*	Fas (TNF receptor superfamily, member 6)	−1.01
*Gadd45a*	Growth arrest and DNA-damage-inducible, alpha	+1.01
*Mapk1*	Mitogen activated protein kinase 1	−1.35
*Mcl1*	Myeloid cell leukemia sequence 1	+1.39
*Pycard*	PYD and CARD domain containing	−1.71
*Xiap*	X-linked inhibitor of apoptosis	+1.26
**1200DSW vs TW**		
*Aifm1*	Apoptosis-inducing factor, mitochondrion-associated 1	−1.02
*Api5*	Apoptosis inhibitor 5	−1.39
*Bag1*	BCL2-associated athanogene	−1.21
*Cideb*	Cell death-inducing DFFA-like effector b	−1.43
*Cycs*	Cytochrome c, somatic	−1.90
*Fas*	Fas (TNF receptor superfamily, member 6)	+1.06
*Gadd45a*	Growth arrest and DNA-damage-inducible, alpha	+1.04
*Mapk1*	Mitogen activated protein kinase 1	−1.14
*Mcl1*	Myeloid cell leukemia sequence 1	+1.04
*Pycard*	PYD and CARD domain containing	−1.54
**SeW vs TW**		
*Aifm1*	Apoptosis-inducing factor, mitochondrion-associated 1	+3.35
*Api5*	Apoptosis inhibitor 5	+2.25
*Bag1*	BCL2-associated athanogene	+4.24
*Casp1*	Caspase 1	+4.21
*Cideb*	Cell death-inducing DFFA-like effector b	+4.49
*Cycs*	Cytochrome c, somatic	+2.53
*Fas*	Fas (TNF receptor superfamily, member 6)	+2.57
*Gadd45a*	Growth arrest and DNA-damage-inducible, alpha	+7.19
*Mapk1*	Mitogen activated protein kinase 1	+5.29
*Mcl1*	Myeloid cell leukemia sequence 1	+4.13
*Pycard*	PYD and CARD domain containing	+1.61
*Xiap*	X-linked inhibitor of apoptosis	+4.90

**Fold-Change** [2∧(−Delta Delta Ct)] is the normalized gene expression [2∧(−Delta Ct)] in the test sample divided by the normalized gene expression [2∧(−Delta Ct)] in the control sample.

### pH and ORP values in the gastric or duodenal fluids in response to TW and DSW infusion

We measured the response of pH and ORP values when the TW or DSW was infused into the stomach (**Figure 1A**). We found changes in ORP (from +600 mV to +360 mV) and pH values (from 7.6 to 3.8) in the outflow of TW fluids through the stomach. We also demonstrated changes in ORP (from +170 mV to +180 mV) and pH values (from 9.7 to 3.9) in the outflow of DSW1200 through the stomach (**Figure 1B**).

To measure optimal pH and ORP values of waters in the duodenum (**Figure 1C**), our results showed the changes in ORP (from +600 mV to +190 mV) and pH values (from 7.4 to 7.8) in the outflow of TW fluids through the duodenum. We also observed changes in ORP (from +165 mV to +170 mV) and pH values (from 8.7 to 7.8) in the outflow of DSW1200 through the duodenum (**Figure 1D**).

### TW and DSW drinking have a similar effect on intestinal oxygenation and motility

**Figure 2:**
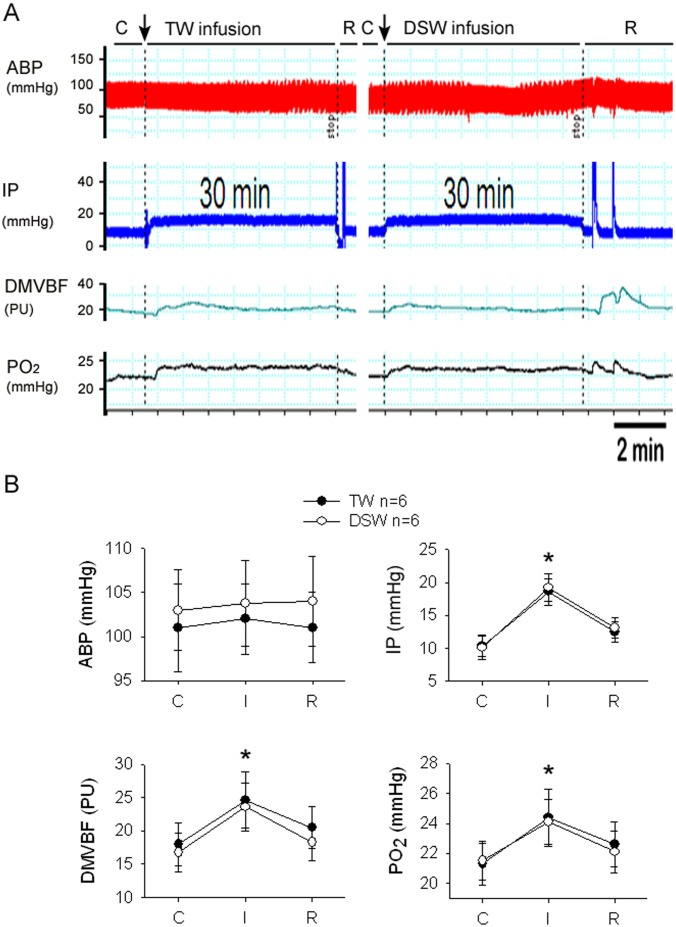
Effect of water infusion on intestinal pressure, microvascular blood flow and oxygen concentration. The typical recordings (A) and statistical data (B) relating to changes in arterial blood pressure (ABP), intestinal pressure (IP), duodenal microvascular blood flow (DMVBF) and partial oxygen concentration in the duodenum in response to tap water (TW) or deep-sea water (DSW) infusion. C = control stage; I = intestinal infusion of TW or DSW; R = recovery stage after infusion. *P<0.05 vs. control stage.

Original values for duodenal pressure, microcirculation, and tissue oxygenation are shown in **Figure 2A**. ABP response did not change before (control stage), during (infusion stage) or after intestinal infusion (recovery stage) in response to TW and DSW. However, in response to TW and DSW infusion, intestinal pressure (from 10.4±1.6 to 18.6±2.0 mmHg in the TW group and from 10.1±1.8 to 19.2±2.1 mmHg in the DSW group), duodenal microvascular blood flow (from 18.0±3.2 to 24.6±4.2 PU in the TW group and from 16.8±2.9 to 23.6±3.6 PU in the DSW group), and PO_2_ (from 21.3±1.4 to 24.4±1.9 mmHg in the TW group and from 21.5±1.3 to 24.1±1.5 mmHg in the DSW group) all significantly increased to a similar degree (*P*<0.05) (**Figure 2B**). After infusion, intestinal pressure immediately returned to its baseline level, whereas duodenal microvascular blood flow and PO_2_ required 2–5 min to return to the baseline level.

### DSW via selenium and not high [Mg^2+^] provides duodenal protection against acid-induced ulcer and apoptosis

**Figure 3:**
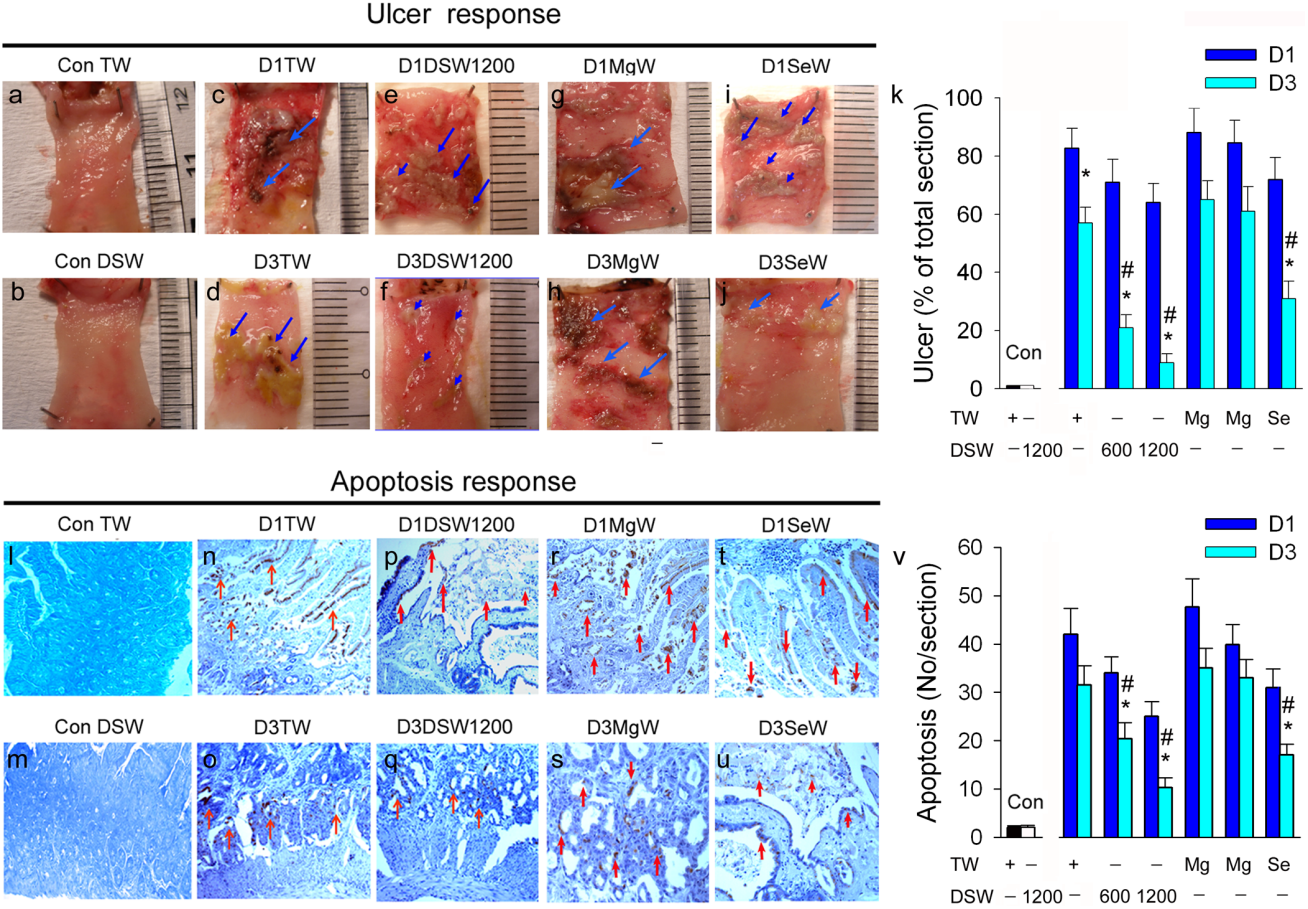
The comparison of different waters consumption on acetic acid-induced duodenal injury. Effect of one-week consumption of tap water (TW), deep-sea water (DSW), magnesium water (MgW) or selenium water (SeW) on acetic acid-induced duodenal ulcers and apoptosis in rats. The ulcer area is indicated with blue arrows (a–j) and the statistical data relating to the ulcers are indicated as k. Apoptosis formation is indicated with red arrows (l–u) and statistical data relating to apoptosis is summarized in°v. Con TW = control group with TW (black bar); Con DSW = control group with DSW1200 (white bar); D1TW = day 1 ulcer with TW; D3TW = day 3 ulcer with TW; D1DSW600 = day 1 ulcer with DSW600; D3DSW600 = day 3 ulcer with DSW600; D1DSW1200 = day 1 ulcer with DSW1200; D3DSW1200 = day 3 ulcer with DSW1200; D1MgW = day 1 ulcer with MgCl_2_ in DDW; D3MgW = day 3 ulcer with MgCl_2_ in DDW; D1SeW = day 1 ulcer with Se in DDW; D3SeW = day 3 ulcer with Se in DDW;. *P<0.05 vs. respective D1 group. #*P*<0.05 vs. D3TW group.

We compared TW, DSW, MgCl_2_ water, and selenium water on acetic acid-induced ulcers (**Figures 3** **a–k**) and apoptosis (**Figures 3** **l–v**). In the control condition, daily consumption of TW (control group) or DSW1200 for one week did not affect the duodenal epithelium in control TW (**Figure 3a**) or DSW1200 groups (**Figure 3b**). In response to acetic acid, the duodenal areas in all six groups were damaged and displayed ulcers. After 24-h acetic acid injury, the D1TW (**Figure 3c**), D1DSW1200 (**Figure 3e**), D1MgW [450 mg/L] (**Figure 3g**), and D1SeW (**Figure 3i**) groups all displayed duodenal ulcers. It seems that DSW600, DSW1200, and Se water has a reduced, but not significantly reduced, tendency to attenuate ulcer area compared with the D1TW group. After 72 h of acetic acid injury, ulcer areas had decreased in all groups (**Figure 3d** in D3TW, **Figure 3f** in DSW1200, **Figure 3h** in D3Mgw, and **Figure 3j** in D3SeW). We found that consuming DSW1200, DSW600, and SeW, but not MgW, significantly reduced the area of acetic acid-induced duodenal ulcers (**Figure 3k**).

Daily consumption of TW or DSW1200 for one week did not induce duodenal apoptosis in the TW group (**Figure 3l**) or DSW1200 group (**Figure 3m**). In response to acetic acid, duodenal areas in all six groups were damaged and displayed duodenal apoptosis. After 24 h of acetic acid injury, D1TW (**Figure 3n**), D1DSW1200 (**Figure 3p**), D1MgW [450 mg/L] (**Figure 3r**), and D1SeW (**Figure 3t**) groups all displayed duodenal apoptosis. It seems that DSW600, DSW1200, and Se water has a reduced, but not significantly reduced, tendency to attenuate apoptosis compared with the D1TW group. After 72 h of acetic acid injury, the appearance of apoptosis decreased in all groups (**Figure 3o** in D3TW, **Figure 3q** in DSW1200, **Figure 3s** in D3Mgw, and **Figure 3u** in D3SeW). We found that consumption of DSW1200, DSW600, and SeW, but not MgW, significantly reduced the area of acetic acid-induced apoptosis (**Figure 3v**).

### DSW attenuates duodenal ulcer area via antioxidant and anti-apoptotic mechanisms

**Figure 4:**
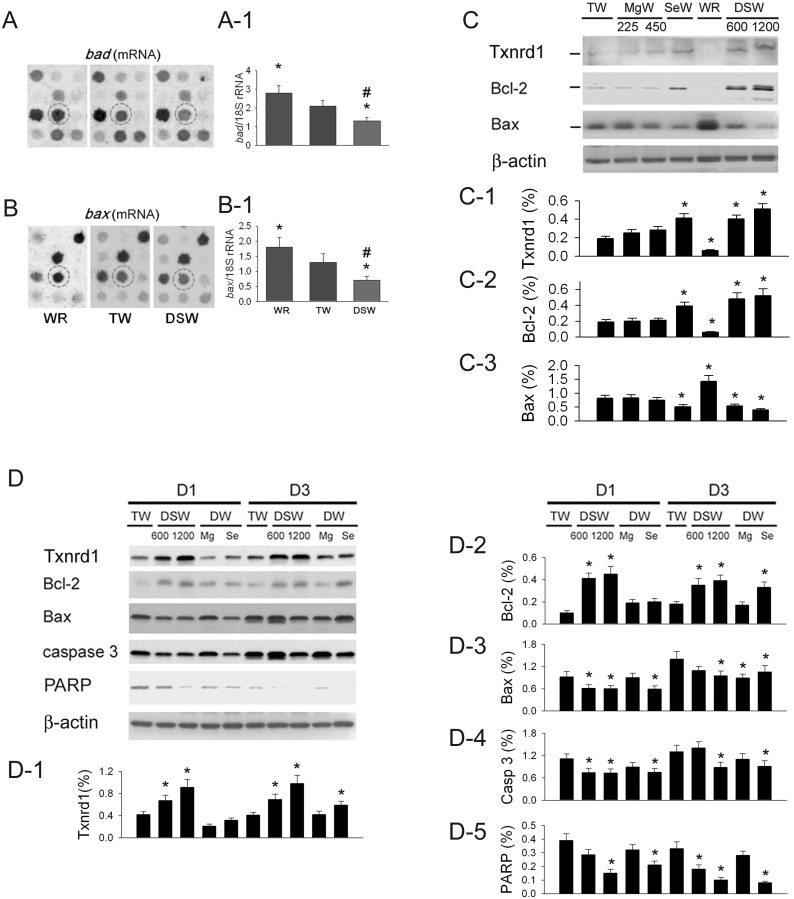
Effect of different water drinking on apoptotic- and antioxidant-related mRNA and protein expression in duodenal tissue. **A:** Duodenal *bad* mRNA (in circle) expression in response to WR, tap water (TW) and DSW1200. The statistical data is shown in **A-1. B**: Duodenal *bax* mRNA (in circle) expression in response to WR, TW, and DSW1200. The statistical data is shown in **B-1. C**: The original data relating to duodenal Txnrd1, Bcl-2, and Bax protein expression in response to TW, MgW with 225 mg/L or 450 mg/L, SeW with selenium 1 mg/100 mL, WR, DSW600, and DSW1200. Statistical data relating to Txnrd1 (**C-1**)**,** Bcl-2 (**C-2**), and Bax (**C-3**) is also demonstrated. **D**: Effect of one week of TW or DSW consumption on the apoptotic- and antioxidant-related protein expression in acetic acid-induced duodenal ulcers. The statistical data relating to Txnrd1 (**D-1**), Bcl-2 (**D-2**), Bax (**D-3**), caspase 3 (**D-4**), and PARP (**D-5**) is indicated. TW = tap water; D1TW = day 1 ulcer with TW; TW = Day 3 ulcer with TW; DSW = deep-sea water; D1DSW = day 1 ulcer with DSW; D3DSW = day 3 ulcer with DSW. MgW = 225 mg/L or 450 mg/L of MgCl_2_ in distilled water (DW); SeW = 1 mg/L of sodium selenite in DW. *P<0.05 vs. respective TW group.

**Figure 4A** shows that duodenal *bad* mRNA expression is different in response to 12 h of WR, 12 h of TW, and DSW1200 ingestion. The results of the statistical analysis indicate that the level of *bad* mRNA expression decreased as follows: WR>TW>DSW1200 (**Figure 4A** **-1**). In **Figure 4B**, duodenal *bax* mRNA expression is shown to be similar to *bad* in response to 12 h of WR, 12 h of TW, and DSW1200 consumption. The data indicated the level of *bax* mRNA expression decreased as follows: WR>TW>DSW1200 (**Figure 4B** **-1**). **Figure 4C** shows the original Western blot of duodenal Txnrd1, Bcl-2, and Bax protein expression in response to one week of TW, Mg water with 225 mg/L or 450 mg/L, selenium water with selenium (sodium selenite) 1 mg/L, DSW600, and DSW1200 consumption and 12 h of WR consumption. We found that DSW1200 and selenium water increased duodenal Txnrd1 (**Figure 4C** **-1**) and Bcl-2 expression (**Figure 4C** **-2**) and depressed Bax expression (**Figure 4C** **-3**). The WR group displayed a significant reduction in Txnrd1 and Bcl-2 and a significant increase in Bax expression compared with the TW group (*P*<0.05). Ingestion of Mg water showed a similar effect to that seen in the TW group.

**Figure 4D** shows the Western blot data of one week of TW, DSW, Mg water or selenium water drinking on apoptotic- and antioxidant-related protein expression in acetic acid-induced duodenal ulcers. These data show that consumption of DSW600, DSW1200 or selenium water, but not Mg water with 225 or 450 mg/L, significantly upregulated Txnrd1 (**Figure 4D** **-1**) and Bcl-2 (**Figure 4D** **-2**) and downregulated Bax **(** **Figure 4D** **-3**), caspase 3 (**Figure 4D-4**), and PARP (**Figure 4D** **-** **5**) in duodenal cells. DSW ingestion provides intestinal protection via the antioxidant and anti-apoptotic mechanisms of selenium but not Mg^2+^.

**Figure 5:**
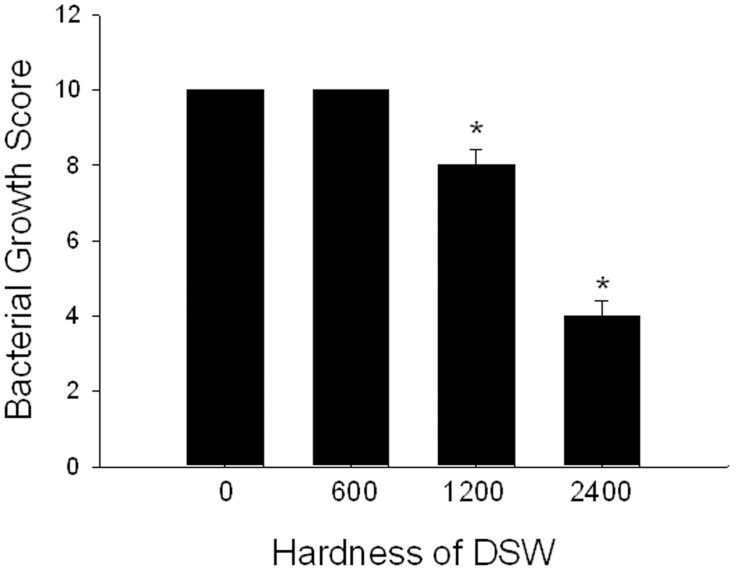
The effect of DSW on *H. pylori* growth. The experimental data are obtained from three independent tests. *P<0.05 vs. hardness at 0.

### DSW displayed anti-bacterial activity in a hardness-dose-dependent manner

**Figure 5** shows that DSW at hardness of 1200 and 2400 inhibited 20% and 60% growth of *H. pylori* strains, respectively. However, DSW at hardness of 600 had no effect on the *H. pylori* growth.

## Discussion

In our *in* *vitro* study, DSW was characterized by higher pH value, lower ORP value, higher concentration of Na^+^, K^+^, Ca^2+^, Mg^2+^ ions and selenium concentration, and higher scavenging H_2_O_2_ and HOCl activity compared with TW. Ingestion of DSW or TW promotes small intestine motility, microcirculation, and temperature to a similar degree. However, the microarray data suggest that ingestion of 600DSW or 1200DSW seems to upregulate antioxidant and anti-apoptotic genes and downregulate apoptotic gene expression, thereby providing antioxidant and anti-apoptotic action in the duodenal epithelium. Our *in* *vivo* data confirm that DSW ingestion attenuates the area of acetic acid-induced duodenal ulcers and apoptosis numbers via the action of selenium to induce Bcl-2 and Txnrd1 expression.

Previous studies have demonstrated that DSW ingestion inhibits endotoxin-induced septic inflammation, infiltration of inflammatory foam cells in the hyperlipidemic aorta [5], and nitric oxide levels in the cataract lens [9], [10], mainly due to its high Mg^2+^ content [27]. DSW consumption lowers the lipid profile, levels of serum thiobarbituric acid-reactive substances, and lipid peroxidation levels in hypercholesterolemic subjects and increases antioxidant enzyme glutathione peroxidase-1 activity [4], [5], [28], possibly due to its high Mg^2+^ and Ca^2+^content. Bathing in a magnesium-rich Dead Sea salt solution improves skin barrier function, enhances skin hydration, and reduces inflammation in atopic dry skin [29]. On the other hand, in a previous study, DSW ingestion decreased inflammatory skin responses in 27 out of 33 patients, decreased inflammatory cell infiltration, IgE, histamine, and inflammatory cytokines [7], [8], due to its increased selenium levels. Selenium, an essential trace element for animals and humans, has been shown to affect the functions of several specific intracellular selenoproteins include glutathione peroxidases and thioredoxin reductases, which have important antioxidant and detoxification functions [25]. These data suggest high Mg^2+^ and selenium concentrations play an important role in the preventive action of DSW and may have therapeutic potential. This is the first study to show that DSW itself can directly scavenge H_2_O_2_ and HOCl amount and upregulate antioxidant (Txnrd1) and anti-apoptotic (Bcl-2) genes and protein expression in the duodenal epithelium through the action of selenium, but not MgCl_2_.

The induction of ROS formation may be caused by toxicity, infection, or ischemia/reperfusion and can initiate oxidative, inflammatory, and apoptotic pathways leading to cell, and tissue damage [11]–[13]. Previous data have found a positive linear correlation between the number of apoptotic cells and the amount of ROS [11]. Induced endogenous or administered exogenous antioxidants may counteract increased oxidative stress [11], [12], [24]. Txnrd1 is a selenoprotein and selenium is a substrate for Txnrd1 [30]. Selenium is an essential micronutrient for humans and animals and selenium deficiency can predispose towards the development of pathological conditions [31]. *Txnrd1*/Txnrd1 maintains cellular redox balance and regulates several redox-dependent processes in the apoptosis pathway [31]. Our data show that DSW and selenium ingestion significantly upregulated duodenal *Txnrd1*/Txnrd1 expression compared with ingestion of TW. Furthermore, we found that WR significantly upregulated *bad* and *bax* mRNA expression, and downregulated *Gsr* (glutathione reductase) and *Sod1* (superoxide dismutase 1, soluble) gene expression, and Txnrd1 protein expression, suggesting decreased antioxidant and anti-apoptotic defense in the duodenal cells. *Sod1*, also known as *CuZn-SOD*, is a ubiquitous family of enzymes that function to efficiently catalyze the dismutation of superoxide anions. Hoffman-Goetz et al. [32] reported that anti-apoptotic Bcl-2, survival HSP70, and antioxidant CuZn-SOD proteins are upregulated in the intestinal lymphocytes of mice after repeated exercise stress. Our data indicate that ingestion of TW and DSW both increase gut motility and hemodynamics to a similar degree. However, DSW ingestion more efficiently activates antioxidant, anti-inflammatory, and anti-apoptotic gene and protein expressions compared with ingestion of TW or WR. These results suggest that minerals or microelements in DSW may confer intestinal protection.

However, increased ROS levels are associated with the loss of mitochondrial membrane potential and the opening of the outer mitochondrial voltage-dependent anion conductance (VDAC) channel [33]. Increased Bax/Bcl-2 ratio triggers Bax translocation to mitochondria to form a VDAC channel for promoting cytochrome c release and caspase 3 activation [33], [34]. We confirm that activated Bax/Bcl-2 ratio, caspase 3, and PARP signaling contributes to acetic acid-induced duodenal ulceration. At baseline, the expression patterns of Bcl-2 and Bax in duodenal cells are affected by the action of selenium but not by MgCl_2_. Ingestion of DSW600, DSW1200, or selenium upregulates Trxnrd1 as well as Bcl-2 expression and downregulates Bax in duodenal cells. Pretreatment with selenium partially blocked cadmium-induced ROS production, mitochondrial membrane potential collapse, cytochrome c release, and caspase activation, and altered Bcl-2 and Bax levels [34]. Selenium also enhanced glutathione peroxidase-1 and Trxnrd1 expression, providing vascular endothelial protection [35]. Our data indicate that DSW or selenium ingestion increases duodenal Trxnrd1 and Bcl-2 protein expression and inhibits apoptosis signaling and number in acetic acid-induced duodenal ulcer. We suggest that selenium in DSW may provide duodenal protection via antioxidant and anti-apoptotic mechanisms.

In patients with atopic eczema/dermatitis syndrome, 6-month or 12-month DSW drinking daily 500 mL (hardness 1000 ppm, Mg 100.0 mg, Na 37.0 mg, Ca 35.5 mg, K 34.5 mg, Zn 2.0 µg, Cu 2.2 µg, I 4.5 µg, P 4.5 µg and Se 0.2 µg) significantly decreased the levels of potassium and toxic minerals like mercury and lead and significantly increased the levels of Se [7] and 1-year DSW drinking reduced IgE and serum cytokines including interleukin-4, -13 and -18 levels [8]. The human subjects drank 1050 mL of desalted DSW (Mg: 395 mg/L, hardness 1410 ppm) daily for 6 weeks via high Mg to improve lipid metabolism and capably to prevent atherosclerosis [4]. Mg was the major mineral in DSW and Mg levels were 50 times [4] or 200 times (our present study) higher than Ca levels. Based on the results of previous studies [1]–[10], it is reasonable to postulate that high osmotic DSW with high Mg levels and hardness have an importantly protective and therapeutic effect in atopic eczema/dermatitis syndrome, cataract, and cardiovascular diseases. However, there are many minerals and trace elements in DSW, such as sulfate, lithium, selenium, molybdenum, silicon, and zinc, etc. Serum Mg level was increased only at the third week by drinking DSW [4]. It is postulated that Mg was not the sole factor in reducing lipids, as other trace elements like selenium may also be involved. It seems that long-term ingestion of DSW is safe to apply as a clinical medicine or functional supplement to cure or prevent some diseases. As to peptic ulcer, there was one paper reported the inhibitory effects of refined DSW on *H. pylori* growth and motility were found [41]. At a hardness of 1000, anti- *H. pylori* activities were observed in most strains. One liter of refined DSW was given daily for 10 days to healthy subjects infected with *H. pylori*. *In* *vivo* anti- *H. pylori* effects were observed in ≥90% of subjects drinking DSW [41]. Our data also found that increased hardness of DSW dose-dependently inhibited *H. pylori* growth (**Figure 5**). These results raised the possibility that the application of DSW can be available for prevention of or as an adjuvant therapy for *H. pylori* infection.

Peptic ulcer disease was a serious cause of global morbidity and mortality until the last decades of the 20th century [36]–[37]. Peptic ulcer disease significantly impairs wellbeing, reduces health-related quality of life, and is associated with high costs for employers and health care systems [38]. Due to the introduction of antacids, histamine H_2_-receptor antagonists, proton pump inhibitors, and the eradication of *H. pylori*, the paradigm of peptic ulcer disease has changed drastically, with a marked decrease in morbidity and mortality [39]–[40]. Despite substantial advances, peptic ulcers remain an important clinical problem, largely because of the increasingly widespread use of NSAIDs and low-dose aspirin. Therefore, acid suppression continues to be an important prevention strategy for NSAID/aspirin/acid-associated gastric and duodenal ulcers and ulcer complications. In the present study, DSW has been shown to provide antioxidant, anti-inflammatory, and anti-apoptotic protection in the duodenal epithelium and attenuate acetic acid-induced duodenal ulcers and apoptosis in the rat model. We suggest that this effect is related to its high selenium content. DSW ingestion may provide a simple and efficient therapeutic and preventive strategy to protect gastrointestinal mucosa against acid or drug-induced gastrointestinal ulcers.

It has been suggested that selenium via ROS and mitochondria linked signal pathway to regulating apoptosis gene expression. Because a concomitant by the generation of ROS, the loss of mitochondrial membrane potential, cytochrome c release, activation of caspase-9, -3 and regulation of Bcl-2 and Bax were observed in damaged cells [42]. Selenium or DSW, the free radical scavengers, may reduce ROS amount, and result in the inhibition of mitochondrial membrane potential collapse, prevention of cytochrome c release, subsequent inhibition of caspase activation and the changed level of Bcl-2 and Bax. Taken together, we concluded that acetic acid-induced apoptosis was mediated by oxidative stress and selenium produced a significant protection against acetic acid-induced apoptosis in intestinal epithelium possibly via ameliorating the mitochondrial dysfunction.

It was reported that female sex steroids may play a role in drug-induced gastroduodenal ulcers by modulating microvascular permeability and mucus secretion [43]. However, in the human studies, the male to female ratio of peptic ulcer disease has become approximately equal (from 1.9 in 1965 to 1.0 in 1981) [44]. In this study, we use a homogeneous population in the gender (all females), the influence of sex hormones or steroids should be equal to each rat.

In summary, the present data suggest that DSW is characterized by high pH value, lower ORP value, higher Mg^2+^ and selenium concentration, and higher antioxidant H_2_O_2_ and HOCl activity. Consumption of DSW downregulates the duodenal expression of oxidant and apoptotic genes, including *bax* and *bad* mRNA expression, and upregulates antioxidant and anti-apoptotic gene expression compared with the TW or restricted water-drinking groups. DSW provides duodenal protection against acid-induced ulcer and apoptosis via antioxidant and anti-apoptotic defense mechanisms due to selenium and not Mg^2+^.
